# Structural identification of riluzole-binding site on human TRPC5

**DOI:** 10.1038/s41421-022-00410-5

**Published:** 2022-07-12

**Authors:** Yaxiong Yang, Miao Wei, Lei Chen

**Affiliations:** 1 Key Laboratory of Biomechanics and Mechanobiology (Beihang University), Ministry of Education, Beijing Advanced Innovation Center for Biomedical Engineering, School of Biological Science and Medical Engineering, Beihang University, Beijing, China; 2 State Key Laboratory of Membrane Biology, College of Future Technology, Institute of Molecular Medicine, Peking University, Beijing Key Laboratory of Cardiometabolic Molecular Medicine, Beijing, China; 3 Peking-Tsinghua Center for Life Sciences, Peking University, Beijing, China; 4 Academy for Advanced Interdisciplinary Studies, Peking University, Beijing, China

**Keywords:** Cryoelectron microscopy, Cell biology

Dear Editor,

Riluzole (Rilutek) is one of the few drugs prescribed in clinic for the treatment of amyotrophic lateral sclerosis^1^. In addition, riluzole shows promise in the treatment of psychiatric disorders and neurodegenerative movement disorders due to its neuroprotective effect^2,3^. It is reported that riluzole can activate the transient receptor potential canonical subfamily member 5 (TRPC5) channel^4^, which is a nonselective calcium-permeant cation channel^5^. TRPC5 is widely expressed in the brain and kidney, and serves as a potential therapeutic target for psychiatric disorders and progressive kidney disease^6^. TRPC5 channel can be activated by diverse stimuli^5^, including external and internal calcium, trivalent lanthanides (La^3+^, Gd^3+^), depletion of intracellular Ca^2+^ stores, reduced extracellular thioredoxin, hypo-osmotic buffer condition, indirect pathways like the stimulation of membrane receptors (receptor activation), and small-molecule activators like Englerin A and riluzole. Activation of TRPC5 has therapeutic potential for the treatment of kidney cancers^6^. Among the activators, riluzole is highly specific for TRPC5 and does not activate other TRP channels, including the closest homolog TPRC isoforms^4^. Although there are reports on the structures of TRPC5 in complex with several inhibitors available^7–9^, how activators, such as riluzole, bind TRPC5 remains unknown, which impedes not only the elucidation of the activation mechanism of TRPC5 but also structure-based drug discovery targeting TRPC5 activation.

Here we purified the truncated functional human TRPC5 (hTRPC5_1–764_) protein^9^ and supplemented the protein with riluzole for cryo-EM sample preparation. Subsequent single particle analysis resolved the structure of hTRPC5 in complex with riluzole at a global resolution of 2.4 Å (Fig. 1a; Supplementary Figs. S1, S2a–c and Table S1), which is the highest among available TRPC5 structures so far^7–9^. The excellent map quality revealed densities of many lipids and ligands, including previously identified diacylglycerol (DAG)^9^ (Supplementary Fig. S2d) and riluzole (Supplementary Fig. S2e), as well as calcium ion inside the voltage sensor-like domain (VSLD)^9^ (Supplementary Fig. S2f). The overall architecture of riluzole-bound hTRPC5 is similar to the apo state or the clemizole (a TRPC5 inhibitor)-bound structures^7,9^, with a root-mean-square deviation (RMSD) of 0.781 Å and 0.682 Å, respectively. hTRPC5 with riluzole bound shows a four-fold symmetric homotetramer with dimensions of 100 Å × 100 Å × 130 Å. Each monomer of hTRPC5 is comprised of an intracellular cytosolic domain (ICD) and a transmembrane domain (TMD) (Fig. 1a), which can be further divided into ion channel pore (Segment 5–Segment 6, S5–S6 for short) and VSLD (S1–S4) (Supplementary Fig. S2d).

**Fig. 1:**
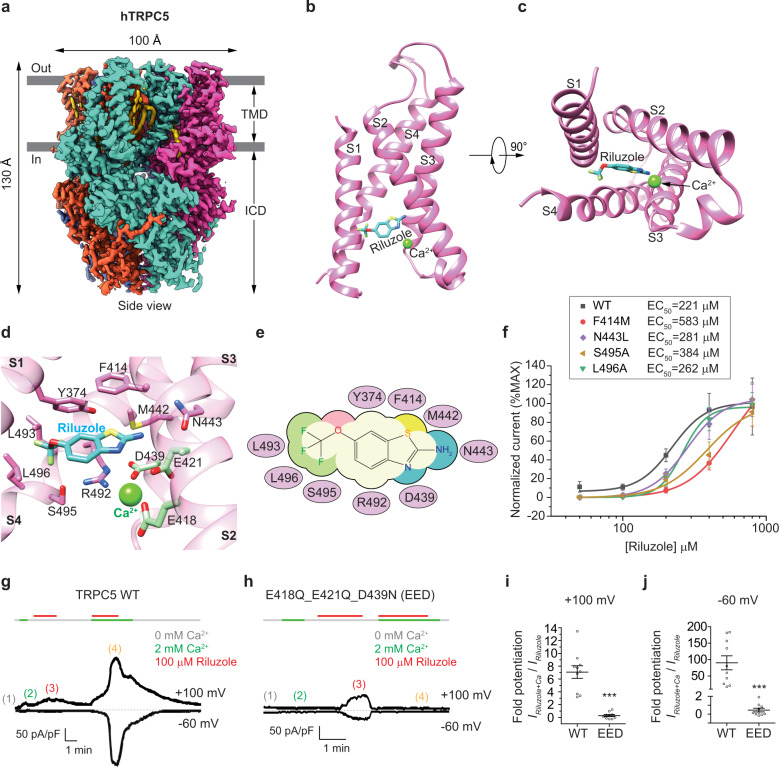
Structure of the hTRPC5 channel in complex with riluzole. **a** The cryo-EM density map of riluzole-bound hTRPC5 shown in side view. Four subunits are colored in hot pink, cyan, orange and blue, respectively. Lipids are colored in yellow. The cell membrane is indicated by gray lines. TMD, transmembrane domain; ICD, intracellular cytosolic domain. **b**, **c** Overview of the riluzole-binding site in hTRPC5. Riluzole is shown as sticks and colored in light sea green. Ca^2+^ ion is shown as a green sphere. Side view (**b**) and bottom view (**c**) are shown. **d** Close-up view of the riluzole-binding site and Ca^2+^-binding site. The main chains of hTRPC5 are shown as cartoons and colored in transparent hot pink. Side chains of interacting residues are shown as sticks. Residues colored in hot pink and D439 interact with riluzole. And residues colored in light green interact with Ca^2+^. **e** Cartoon representation of the interaction between riluzole and hTRPC5. Interacting residues are labeled in the purple ovals. **f** The activation effects of riluzole on various hTRPC5 mutants, measured by electrophysiological recordings under 0 mM extracellular Ca^2+^ at −100 mV. Representative traces are shown in Supplementary Fig. S3. Recordings from at least 10 cells were analyzed for each mutant. Data were fitted by dose-response curves and were normalized to ‘max currents’ (the maximal response produced by the drug). Data are shown as means ± SEM. **g**, **h** Ca^2+^-dependent potentiation of riluzole-activated TRPC5 currents of wild-type (WT) (**g**) and TRPC5_E418Q_E421Q_D439N mutant (EED) (**h**). TRPC5 currents were elicited by 2 s voltage ramps from −60 mV to +100 mV, applied at 0.33 Hz from a holding potential of −60 mV. The currents at +100 mV (the steady currents of 500 ms step of +100 mV at end of ramp, top trace) and −60 mV (the steady currents of 100 ms step of −60 mV before ramp, bottom trace) are plotted. Stimuli of 2 mM extracellular Ca^2+^ (marked as green lines) and 100 μM riluzole (marked as red lines) were individually or synergistically applied to cells. Basal condition of 0 mM extracellular Ca^2+^ is marked as gray lines. Representative ramp traces at basal state (0 mM Ca^2+^, marked with 1 and colored in gray), 2 mM Ca^2+^ (2, in green), 100 μM riluzole in 0 mM Ca^2+^ (3, in red), and 100 μM riluzole in 2 mM Ca^2+^ (4, in yellow) are shown in Supplementary Fig. S4 for comparison. **i**, **j** Statistical summaries of the fold potentiation of riluzole-activated current at +100 mV (**i**) or −60 mV (**j**) in 2 mM Ca^2+^ external relative to 0 mM Ca^2+^ external. *n* = 10 cells for each column. Data are shown in means ± SEM. Two-tailed unpaired Student’s *t-*test was calculated for (**i**) and (**j**) with criteria of significance; ***P < 0.001.

Riluzole binds inside a pocket of VSLD and is adjacent to the Ca^2+^ ion density with the distance of 4 Å (Fig. 1b, c). Several residues on S1, S2, S3, and S4 are closed to the ligand density of riluzole, indicating a direct interaction between the drug and the channel (Supplementary Fig. S2f). Specifically, Y374 on S1 and F414 on S2 form π–π stacking interaction with benzothiazole ring of riluzole. R492 on S4 forms a cation-π interaction with the benzothiazole ring of riluzole. D439 on S3 and S495 on S4 form hydrogen bonding (H-bond) with the amine and trifluoromethoxy of riluzole, respectively. N443 on S3 shows hydrophobic interaction with the amine of riluzole. M442 on S3, L493 on S4, and L496 on S4 may form Van der Waals interaction with riluzole (Fig. 1d–e). To elucidate the contribution of these residues to the binding of riluzole, we mutated them into alanines or their counterparts in hTRPC3/6/7. These mutants, including F414M, N443L, S495A, and L496A, were previously found to have proper tetramer assembly, unaltered surface expression, and robust calcium influx^9^. To avoid the interference of Ca^2+^ modulation, HEK293 cells expressing hTRPC5 mutants were recorded in 0 mM extracellular Ca^2+^ solution. We found that all these mutations decrease the potency of riluzole to some extent (Fig. 1f; Supplementary Fig. S3, and Table S2). Notably, the corresponding residue of F414 in hTRPC5 is Met in hTRPC1/3/6/7, and N443 in hTRPC5 is Leu in hTRPC3/6/7. Both F414M and N443L decrease the potency of riluzole towards hTRPC5 (Fig. 1f), in agreement with the selectivity of riluzole between hTRPC5 and hTRPC3/6/7^4^.

Riluzole-binding site is close to a calcium ion. We wonder if there is any relationship between riluzole activation and calcium activation. The Ca^2+^ ion in VSLD interacts with E418 on S2, E421 on S2, and D439 on S3 (Fig. 1d), and has been reported to mediate calcium activation of TRPC5^9^. hTRPC5 triple mutant (E418Q, E421Q, and D439N, the EED mutant) almost totally abolished the activation of TRPC5 by 14 mM extracellular calcium^9^ (Supplementary Fig. S4a–c). However, the potency of riluzole on this mutant remains unchanged compared to wild-type hTRPC5 (WT) in the presence of 0 mM extracellular Ca^2+^ (Supplementary Fig. S4d). To further study whether calcium binding in this site would affect the activation of riluzole, we carried out electrophysiological experiments to record the hTRPC5 currents under three conditions: 2 mM extracellular Ca^2+^; 100 μM riluzole in 0 mM extracellular Ca^2+^; 100 μM riluzole in 2 mM extracellular Ca^2+^. 2 mM Ca^2+^ or 100 μM riluzole in 0 mM extracellular Ca^2+^ could elicit small hTRPC5 currents. However, the hTRPC5 currents were potentiated by several folds by 100 μM riluzole in 2 mM extracellular Ca^2+^ (Fig. 1g and Supplementary Fig. S4e). In contrast, although 100 μM riluzole could elicit currents in EED mutant in the absence of extracellular Ca^2+^, the presence of 2 mM extracellular Ca^2+^ failed to further potentiate the riluzole-activated current **(**Fig. 1h and Supplementary Fig. S4f). Statistical analysis showed that the riluzole-activated hTRPC5 currents were potentiated ~7-fold at +100 mV and ~90-fold at −60 mV in 2 mM Ca^2+^ relative to 0 mM Ca^2+^. In contrast, the riluzole-activated currents in EED mutant in 2 mM Ca^2+^ were 0.3-fold at +100 mV and 0.5-fold at −60 mV relative to 0 mM Ca^2+^ (Fig. 1i, j). These results comprehensively suggest the synergistic effect between riluzole and the nearby calcium on the activation of TRPC5. This is also in agreement with that the EC_50_ of riluzole is around 210 μM in the absence of extracellular Ca^2+^ at −100 mV (Fig. 1f and Supplementary Table S2) but decreased to 20.7 μM in the presence of 1.5 mM extracellular Ca^2+^ at −100 mV^4^. Besides the Ca^2+^, La^3+^ was reported to potentiate the riluzole-activated TRPC5 currents^4^, but the actual effectiveness is much weaker than expected. The outward currents at +100 mV have no significant change after La^3+^ stimuli. And the La^3+^-dependent potentiation of inward current at −100 mV is < 2-fold^4^. Mutagenesis studies suggest that the La^3+^-binding site locates at the extracellular loop of S5–S6^5^. The different binding sites and weak synergistic effect infer that riluzole and La^3+^ activate the TRPC5 by different mechanisms. In contrast, both riluzole and Ca^2+^ bind inside VSLD and exhibit a remarkable synergistic effect, suggesting that they share a converged activation mechanism.

The ion permeation pathway of riluzole-bound hTRPC5, lined by pore helices, pore loops, and S6 of four protomers (Supplementary Fig. S5a), is constricted at the lower gate formed by I621, N625, and Q629, similar to the apo state and clemizole-bound state (Supplementary Fig. S5b), suggesting that our riluzole-bound structure is in a non-conductive closed state, which correlates with the relatively low open probability of TRPC5 upon riluzole stimulation (*NP*_*O*_ is around 0.4 at −80 mV)^4^. Activators with higher efficacy or gain-of-function mutations might further increase the *P*_*O*_ to stabilize the open-state structure of TRPC5.

We previously reported the structure of hTRPC5 in complex with an inhibitor clemizole^9^. Both clemizole and riluzole are benzothiadiazine derivatives, and bind to the same pocket inside VSLD. The structural comparison showed that the binding pocket of clemizole and riluzole are largely overlapped (Supplementary Fig. S5c). Further analyses on the two structures reveal that their main chains are almost superposable, with a RMSD of 0.682 Å (Supplementary Fig. S5c). All the riluzole-interacting residues are involved in clemizole-binding (Supplementary Fig. S5d). However, because clemizole is larger than riluzole, clemizole interacts with more residues in VSLD than riluzole, including P659, Y446, E418, and G417. These structural observations suggest a competitive binding behavior and are in agreement with previous electrophysiological results^10^.

The synergistic effect between riluzole and Ca^2+^ on hTRPC5 activation is akin to that observed for icilin and Ca^2+^ on the activation of the TRPM8 channel^11^. Icilin is an agonist of TRPM8 and binds inside the VSLD^11^. The binding of icilin and Ca^2+^ in VSLD induces the structural changes of the lower portion of S4 from α helix to 3_10_ helix, which further drive the opening of the TRPM8 channel^11^ (Supplementary Fig. S6a, b). Notably, another TRPM8 agonist WS-12 also binds inside VSLD but failed to drive the conformational change of VSLD and subsequent opening of the channel^11^ (Supplementary Fig. S6a, b). In addition, TRPM8 antagonists, such as AMTB and TC-I 2014, all bind inside the VSLD^12^ (Supplementary Fig. S6a, c). The larger chemical groups of antagonists (molecular weight of AMTB: 431.0 Da and TC-I 2014: 467.4 Da versus WS-12: 289.4 Da and icilin: 311.3 Da) may prevent the activating conformational change of S4 of VSLD and stabilize the closed state of TRPM8^12^ (Supplementary Fig. S6d).

Given the similar structural features of TMD between TRPM8 and TRPC5 and their conserved calcium binding sites in VSLD, we speculate TRPC5 shares a similar activation mechanism to TRPM8. Riluzole might behave like WS-12, an agonist of TRPM8, which has low efficacy, activates the channel to some level and does not prevent the activating conformational changes of VSLD. In contrast, the bulkier clemizole (molecular weight of clemizole: 325.8 Da vs riluzole: 234.2 Da) behaves like AMTB, an antagonist of TRPM8, which also binds inside VSLD but blocks the conformational changes essential for channel activation (Supplementary Fig. S6e).

Besides TRPC5, riluzole is also reported to activate the small conductance, Ca^2+^-activated K^+^ channel (SK channel) in cultured hippocampal neurons and in HEK293 cells expressing recombinant SK2 channels. The crystal structure of SK2 C-terminal domain–calmodulin (CaM)–riluzole complex was reported^13^. The structure revealed that riluzole could bind inside a pocket situated at the intracellular interface of SK2–CaM channel complex^13^ (Supplementary Fig. S7a–c). Although the riluzole-binding residues are different between hTRPC5 and SK2 (Fig. 1d and Supplementary Fig. S7c), the chemical environments of their binding pockets share some similarities (Supplementary Fig. S7d–g). These two pockets exhibit similar negatively-charged surface but different hydrophobic patches. In detail, both pockets exhibit highly negatively-charged surface at the mouth, binding to the positively-charged amine functional group of riluzole. Whereas inner spaces of the two pockets are less negatively-charged (Supplementary Fig. S7d, e). The hydrophobicity of riluzole-binding pocket seems to be less important for riluzole binding, since the inner space of riluzole-binding pocket in TRPC5 is highly hydrophilic, which contrasts with the highly hydrophobic surface of the inner pocket of the SK2–CaM complex (Supplementary Fig. S7f, g). In addition to SK2 and hTRPC5, riluzole has a wide range of effects on multiple ion channels and receptors, including Na_V_, K_V_, Ca_V_, and ligand-gated neurotransmitter receptors^14^. The diversity of riluzole-binding sites might be due to the low molecular weight (234.2 Da) and small van der Waals volume (164 Å^3^) of the drug.

In conclusion, our structure of hTRPC5 in complex with riluzole reveals that the riluzole binds inside the VSLD. The binding site of riluzole overlaps with some of the hTRPC5 inhibitors such as clemizole^9^, and TRPC4 inhibitors such as pyridazinone-based small molecules^15^, suggesting the VSLD is a key regulatory module for TRPC channel activity and a hot spot for the binding of small molecule gating modifiers. Moreover, we found riluzole-binding site is close to the activating Ca^2+^ ion-binding site in VSLD. More importantly, riluzole and calcium display a synergistic effect on the activation of hTRPC5. Our studies provide insights into the gating mechanism of hTRPC5 and how riluzole impacts its function.

## Supplementary information

Supplementary information

## Data Availability

Density maps are deposited at the Electron Microscopy Database (accession codes: EMD-32436) and protein coordinates are deposited at the Protein Data Bank (PDB code: 7WDB).
