# Supplementary material for: Structural identification of riluzole-binding site on human TRPC5
Source: Cell Discov. 2022 Jul 12;8:67. doi: 10.1038/s41421-022-00410-5 (PMC9276808; doi:10.1038/s41421-022-00410-5)
Supplement: Supplementary file 1 — Supplementary information [file 41421_2022_410_MOESM1_ESM.pdf]

Supplementary Materials for

Structural identification of riluzole binding site on human TRPC5

Yaxiong Yang [1#], Miao Wei [2#], Lei Chen [2,3,4*]

[1] Key Laboratory of Biomechanics and Mechanobiology (Beihang University), Ministry of Education, Beijing Advanced Innovation Center for Biomedical Engineering, School of Biological Science and Medical Engineering, Beihang University, Beijing 100083, China.

[2] State Key Laboratory of Membrane Biology, College of Future Technology, Institute of Molecular Medicine, Peking. University, Beijing Key Laboratory of Cardiometabolic Molecular Medicine, Beijing 100871, China.

[3] Peking-Tsinghua Center for Life Sciences, Peking University, Beijing 100871, China.

[4]Academy for Advanced Interdisciplinary Studies, Peking University, Beijing 100871, China.

[#] These authors contribute equally to this work.

*Corresponding author: Lei Chen, chenlei2016@pku.edu.cn

This file includes:

Materials and Methods

Additional references

Supplementary Figs. S1 to S7

Supplementary Tables S1 to S2

Materials and Methods

Key Resources Table				
**Reagent type (species) or resource**	**Designation**	**Source or reference**	**Identifiers**	**Additional information**
Gene (Homo sapiens)	hTRPC5	Song et al., 2021. PMID: 33683200	NCBI Reference sequence: NM_012471	Provided by Dr. Xiaolin Zhang, Dizal Pharmaceutical Company
Recombinant DNA reagent	hTRPC5 WT, F414M, N443L, S495A, and L496A	Song et al., 2021. PMID: 33683200		Subcloned into a pCDNA3.1 vector with no tag.
Recombinant DNA reagent	hTRPC5$_{1-764}$	Song et al., 2021. PMID: 33683200		Subcloned into a pBM Bacmam vector with N terminal GFP-MBP tag
Cell line (FreeStyle 293-F)	FreeStyle 293-F	Thermo Fisher Scientific	R79007	RRID:CVCL_D603
Cell line (Sf9)	Sf9	Thermo Fisher Scientific	12659017	RRID:CVCL_0549
Chemical compound, drug	riluzole	TargetMol	T0349	
Software, algorithm	Gctf_v1.18	K. Zhang. (2016). PMID: 26592709		
Software, algorithm	Gautomatch v0.56	K. Zhang, MRC LMB (https://www2.mrc-		

		lmb.cam.ac.uk/research/locally-developed-software/zhang-software/)		
Software, algorithm	RELION-3.1	Zivanov J, et al. 2020. PMID: 32148853		
Software, algorithm	PHENIX 1.18.1-3865	P. D. Adams et al. 2010. PMID: 20124702		
Software, algorithm	HOLE2 v2.2.005	Smart OS, et al. 1996. PMID: 9195488		
Software, algorithm	Chimera-1.13	Pettersen et al. 2004. PMID:15264254		
Software, algorithm	ChimeraX 1.0	Goddard TD, et al. 2018. PMID: 28710774		

Cell culture

Sf9 cells (Thermo Fisher Scientific) and HEK293F cells (Thermo Fisher Scientific) were cultured in Sf-900 III SFM medium (Gibco) at 27 °C and in Free Style 293 medium + 1% FBS at 37 °C, respectively. HEK293F cells cultured in SMM-293TI medium + 1% FBS at 37 °C were used for baculoviral infection and protein purification. All these cell lines were free of mycoplasma contamination, tested by MycoBlue Mycoplasma Detector assay (Vazyme, D101-02).

Fluorescence-detection size-exclusion chromatography (FSEC)

A home-made BacMam vector with N terminal GFP-MBP tag[1] was used for the cloning of $hTRPC5_{1-764}$. HEK293F cells were transfected by using PEI. Cells were harvested after 48 hours by centrifuge at 4,000 rpm for 10 min at 4 °C, solubilized by 1% (w/v) LMNG, 0.1% (w/v) CHS in TBS buffer (20 mM Tris-HCl, pH 8.0 at 4 °C, and 150 mM NaCl) with 1 ug/ml aprotinin, 1 ug/ml leupeptin, 1 ug/ml pepstatin for 30 min at 4 °C. Cell lysates were centrifuged at 40,000 rpm for 30 min. Supernatants were collected and loaded onto superpose 6 increase (GE healthcare) for FSEC analysis before large-scale expression.

Protein expression and purification

The BacMam expression system used for large-scale expression of $hTRPC5_{1-764}$ was according to our previous studies[2,3]. In Brief, construct of GFP-MBP-$hTRPC5_{1-764}$ in home-made BacMam vector was transformed into DH10Bac E. coli cells to generate bacmid. Baculovirus was harvested about 1-week post transfection of bacmid into Sf9 cells cultured in Sf-900 III SFM medium (Gibco) at 27 °C. P2 baculovirus was generated by the Bac-to-bac system and was added to HEK293F cells at a ratio of 1:12.5 (v/v) when cell density was around 2.0×10^6/ml. Cells were then cultured in SMM 293T-I medium (Sino Biological Inc.) supplemented with 1% FBS under 5% CO2 in a shaker at 130 rpm at 37℃ for 12 hours before adding 10 mM sodium butyrate. After that, cells were transferred to a 30 °C incubator for 2 days and were harvested 48 h post-infection. Cell pellets were collected after rinsing twice by TBS buffer and stored at -80 °C for further purification.

Cell pellets were resuspended in TBS buffer supplemented with 1% (w/v) LMNG, 0.1% (w/v) CHS, 1 mM dithiothreitol (DTT), 1 mM phenylmethanesulfonylfluoride (PMSF), and protease inhibitors, including 1 μg/ml aprotinin, 1 μg/ml leupeptin, 1 μg/ml pepstatin at 4 °C. The mixture was incubated at 4 °C for 1 h and ultra-centrifuged at 40,000 rpm for 1 h in Ti45 rotor (Beckman). Supernatants were collected and loaded onto 7 ml MBP resin. Wash buffer 1 (wash buffer 2 + 1mM ATP + 10 mM $MgCl_2$) and wash buffer 2 (TBS + 40 μM glycol-diosgenin (GDN) + 0.005 mg/ml SPLE + 1 mM DTT) were used to rinse the resin subsequently. Proteins were eluted with 80 mM maltose in wash buffer 2. The eluate was digested with H3C protease at 4 °C overnight to cleave the tags and then concentrated by a 100-kDa cut-off concentrator (Millipore). Cleaved hTRPC5 protein was further purified by size exclusion chromatography (Superose-6, GE Healthcare) in buffer containing TBS, 40 μM GDN, 0.005 mg/ml SPLE, and 1 mM Tris (2-carboxyethyl) phosphine (TCEP). The peak fractions corresponded to the tetrameric TRPC5 channel and were collected for cryo-EM sample preparation. Proteins were concentrated to $A_{280} = 1.0$ with estimated concentration of 0.7 mg/ml.

Cryo-EM sample preparation and data collection

Purified protein was mixed with 100 μM Ca^{2+} and 500 μM riluzole. After incubation with ligands for 30 min on ice, the mixtures were ultra-centrifugated at 25,000 rpm for 30 min in TLA55 rotor (Beckman) and the supernatants were used for cryo-EM sample preparation. Aliquots of 2.5 μl mixture were placed on graphene oxide (GO)-

grids according to the previous report[4]. Grids were blotted at 100% humidity and flash-frozen in liquid ethane cooled by liquid nitrogen using Vitrobot Mark I (FEI). Then grids were transferred to a Titan Krios (FEI) electron microscope that was equipped with a Gatan GIF Quantum energy filter and operated at 300 kV accelerating voltage. Image stacks were recorded on a Gatan K2 Summit direct detector in super-resolution counting mode using SerialEM at a nominal magnification of 165,000 × (calibrated pixel size of 0.821 Å/pixel), with a defocus ranging from -1.5 to -2.0 μm. Each stack of 32 frames was exposed for 7.1s, with a total dose of about 50 e^-/ Å ^{2} and a dose rate of 8 e^-/pixel/s on the detector.

Image processing

The collected image stacks were firstly motion corrected by MotionCor2[5]. Then the micrographs with high quality were manually selected. The GCTF was used for CTF estimation[6]. Particles were auto-picked using Gautomatch (kindly provided by Kai Zhang) based on the projection of hTRPC5 map (EMDB: EMD-30987). After three-rounds reference-free two-dimensional (2D) classification, particles in classes with good features were selected for global search three-dimensional (3D) classification with previous hTRPC5 map (EMDB: EMD-30987) low-pass filtered to 15 Å as the initial model using RELION-3.1 with *C1* symmetry[7,8]. Each of the last 6 iterations in global 3D classification was performed multi-reference local angular search 3D classification. The multi-reference models were generated with hTRPC5 map (EMDB: EMD-30987) low-pass filtered to 8 Å, 15 Å, 25 Å, 35 Å, respectively.

Particles in good classes with clearly visible α-helices in TMD were selected for further two-rounds random-phase 3D classification. Random-phase models beyond 40 Å and 30 Å were generated from the hTRPC5 map (EMDB: EMD-30987). Finally, particles in the good class were subjected to local search homogeneous refinement using *C4* symmetry tandem with CTF refinement. Reported resolutions are based on the gold-standard Fourier shell correlation (FSC) 0.143 criterion after correction of masking effect[9]. The map was sharpened with a *B* factor of -50.

Model building, refinement, and validation

The previously reported TRPC5 (PDB: 7E4T) was used as the initial model and docked into the riluzole-bound hTRPC5 map in Chimera[10]. The fitted model was manually adjusted in COOT[11]. The ligands models were generated using elbow module[12] in PHENIX[13]. Riluzole was manually docked into densities and refined using COOT. The model was further refined against the corresponding map with PHENIX[13].

Whole cell recording

HEK293F cells were transfected with the 900 ng cDNA of non-tagged full length hTRPC5 in pcDNA3.1 vector by PEI. 100 ng cDNA of home-made GFP-MBP vector was co-transfected into HEK293F cells for picking up positive cells. Cells were used after 36 hours and were plated on cover glasses for whole cell recording at room temperature using an Axopatch 200B amplifier (Axon Instruments). Electrodes were

pulled by a horizontal microelectrode puller (P-100, Sutter Instruments) and heat-polished by a pipette microforge to adjust the resistances around 2-5 MΩ. Currents were recorded at 2 kHz by the low-pass filter. For testing the potency of riluzole on hTRPC5, currents were recorded in gap-free mode at a holding potential of -100 mV. The bath solution of 0 mM Ca^{2+} contained (in mM): 140 NaCl, 5 CsCl, 10 EGTA, 1MgCl$_2$, 10 glucose and 10 HEPES (pH7.4 adjusted with NaOH). The bath solution of 14 mM Ca^{2+} in free state contained (in mM): 24 CaCl$_2$, 104 NaCl, 5 CsCl, 10 EGTA, 1 MgCl$_2$, 10 glucose and 10 HEPES (pH7.4 adjusted with NaOH). The internal solution contained (in mM): 110 CsMe, 25 CsCl, 2 MgCl$_2$, 0.5 EGTA and 30 HEPES (pH 7.4 adjusted with CsOH). For experiments of Ca^{2+}-dependent potentiation of riluzole-activated hTRPC5 current, currents were elicited by repetitive 2 s voltage ramps from -60 mV to +100 mV, applied at 0.33 Hz from a holding potential of -60 mV. A 500 ms step of +100 mV was followed at end of each ramp. The steady currents at +100 mV and -60 mV were plotted for presentation. The bath solution of 0 mM Ca^{2+} contained (in mM): 150 NaCl, 4 KCl, 3 MgCl$_2$, 10 HEPES and 10 glucose (pH 7.4 adjusted with NaOH). The bath solution of 2 mM Ca^{2+} contained (in mM): 150 NaCl, 4 KCl, 2 CaCl$_2$, 1 MgCl$_2$, 10 HEPES and 10 glucose (pH 7.4 adjusted by NaOH). The internal solution contained (in mM): 150 CsCl, 2 MgCl$_2$, 0.36 CaCl$_2$, 1 EGTA and 10 HEPES (pH 7.3 adjusted with CsOH, yielding a free [Ca^{2+}] of around 100 nM at 25 °C using MaxChelator (https://somapp.ucdmc.ucdavis.edu/pharmacology/bers/maxchelator/CaMgATPEGTA-Ts.htm)).

Quantification and statistical analysis

The local resolution map was calculated using RELION-3.1[8]. The pore radius was calculated using HOLE[14]. RMSD was calculated using PyMOL in all-atom model.

To calculate the potency of riluzole, hTRPC5 currents elicited by different concentrations of riluzole were firstly fitted by the dose-response curve as below to obtain a "Max current" for each hTRPC5 variant.

$$y = Min + (Max - Min)/(1 + 10^{(LogEC_{50} - x) \times HillSlope})$$

Then all data points in each hTRPC5 variant were normalized to the "Max current", and were fitted by dose-response curve again for presentation. Electrophysiological data were analyzed by Clampfit, GraphPad Prism and OriginPro. Data were shown in mean±SEM. 5 or more than 5 cells were analyzed for all electrophysiological experiments. Unpaired Student's *t*-test (two-tailed with criteria of significance) was performed to compare two groups. Statement of Significance: **, $p<0.01$; ***, $p<0.001$ and *n.s.* denotes 'not significant' ($p>0.05$).

Additional References

1 Guo, W., Wang, M. & Chen, L. A co-expression vector for baculovirus-mediated protein expression in mammalian cells. *Biochemical and biophysical research communications* **594**, 69-73, doi:10.1016/j.bbrc.2022.01.056 (2022).

2 Tang, Q. *et al.* Structure of the receptor-activated human TRPC6 and TRPC3 ion channels. *Cell Res* **28**, 746-755, doi:10.1038/s41422-018-0038-2 (2018).

3 Song, K. *et al.* Structural basis for human TRPC5 channel inhibition by two distinct inhibitors. *Elife* **10**, doi:10.7554/eLife.63429 (2021).

4 Phulera, S. *et al.* Cryo-EM structure of the benzodiazepine-sensitive α1β1γ2S tri-heteromeric GABA(A) receptor in complex with GABA. *Elife* **7**, doi:10.7554/eLife.39383 (2018).

5 Zheng, S. Q. *et al.* MotionCor2: anisotropic correction of beam-induced motion for improved cryo-electron microscopy. *Nat Methods* **14**, 331-332, doi:10.1038/nmeth.4193 (2017).

6 Zhang, K. Gctf: Real-time CTF determination and correction. *J Struct Biol* **193**, 1-12, doi:10.1016/j.jsb.2015.11.003 (2016).

7 Zivanov, J. *et al.* New tools for automated high-resolution cryo-EM structure determination in RELION-3. *Elife* **7**, doi:10.7554/eLife.42166 (2018).

8 Zivanov, J., Nakane, T. & Scheres, S. H. W. Estimation of high-order aberrations and anisotropic magnification from cryo-EM data sets in RELION-3.1. *IUCrJ* **7**, 253-267, doi:10.1107/S2052252520000081 (2020).

9 Chen, S. *et al.* High-resolution noise substitution to measure overfitting and validate resolution in 3D structure determination by single particle electron cryomicroscopy. *Ultramicroscopy* **135**, 24-35, doi:10.1016/j.ultramic.2013.06.004 (2013).

10 Pettersen, E. F. *et al.* UCSF Chimera--a visualization system for exploratory research and analysis. *Journal of computational chemistry* **25**, 1605-1612, doi:10.1002/jcc.20084 (2004).

11 Emsley, P., Lohkamp, B., Scott, W. G. & Cowtan, K. Features and development of Coot. *Acta Crystallogr D Biol Crystallogr* **66**, 486-501, doi:10.1107/s0907444910007493 (2010).

12 Moriarty, N. W., Grosse-Kunstleve, R. W. & Adams, P. D. electronic Ligand Builder and Optimization Workbench (eLBOW): a tool for ligand coordinate and restraint generation. *Acta Crystallogr D Biol Crystallogr* **65**, 1074-1080, doi:10.1107/s0907444909029436 (2009).

13 Adams, P. D. *et al.* PHENIX: a comprehensive Python-based system for macromolecular structure solution. *Acta Crystallogr D Biol Crystallogr* **66**, 213-221, doi:10.1107/S0907444909052925 (2010).

14 Smart, O. S., Neduvelil, J. G., Wang, X., Wallace, B. A. & Sansom, M. S. HOLE: a program for the analysis of the pore dimensions of ion channel structural models. *Journal of molecular graphics* **14**, 354-360, 376, doi:10.1016/s0263-7855(97)00009-x (1996).

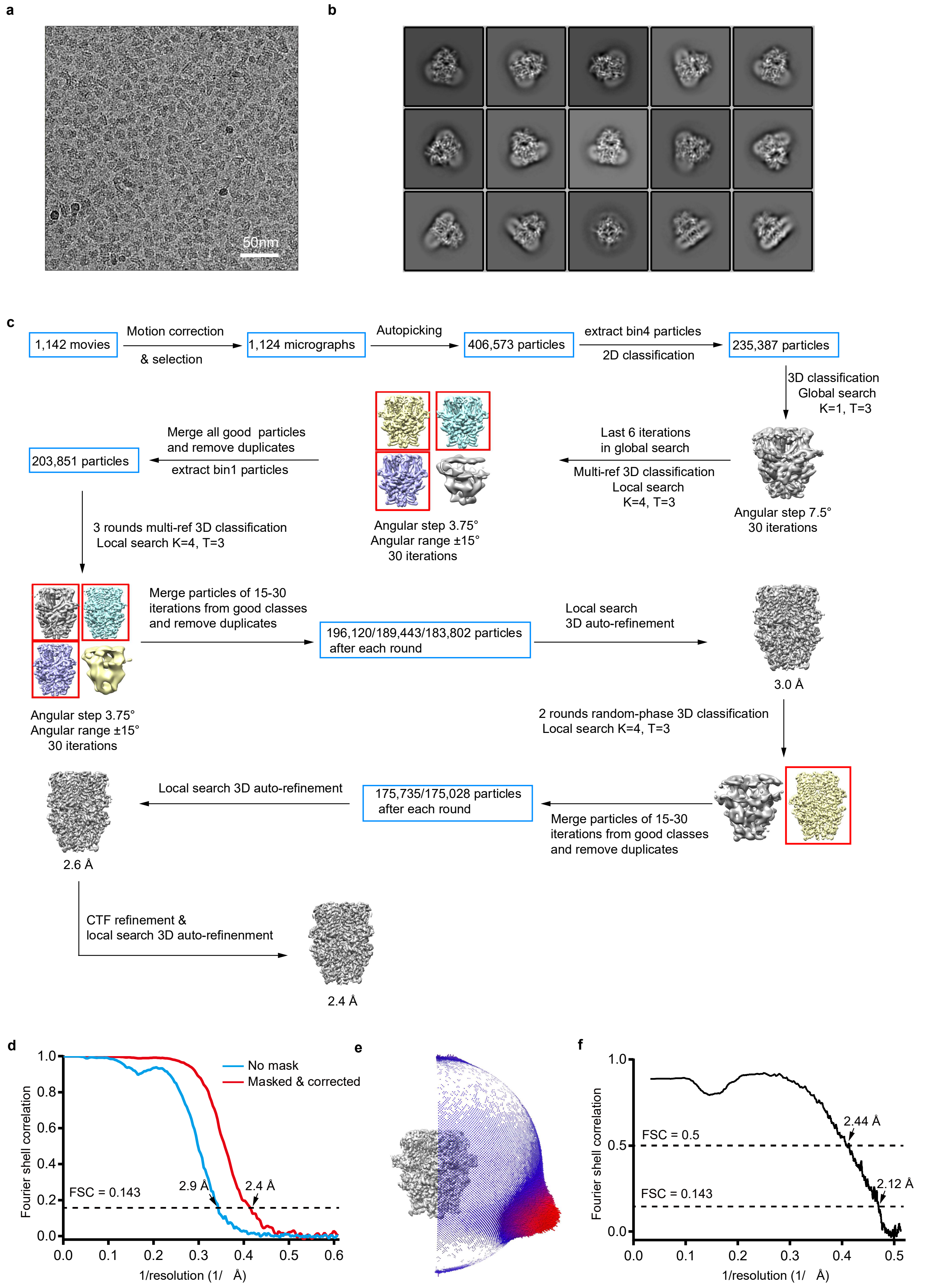

Supplementary Fig. S1. Cryo-EM image analysis of riluzole-bound human TRPC5. a Representative raw micrograph recorded on K2. **b** Representative 2D class averages of riluzole-bound hTRPC5. **c** Flowchart for cryo-EM data processing of riluzole-bound hTRPC5. **d** Fourier shell correlation (FSC) curves of the two independently refined maps for unmasked (blue curve, 2.9 Å) and masked & corrected (red curve, 2.4 Å). Estimation of resolution was based on the criterion of FSC 0.143 cut-off. **e** Angular distribution of riluzole-bound hTRPC5 calculated by RELION-3.1. **f** FSC curve of the refined model versus EM map.

Fig. S1

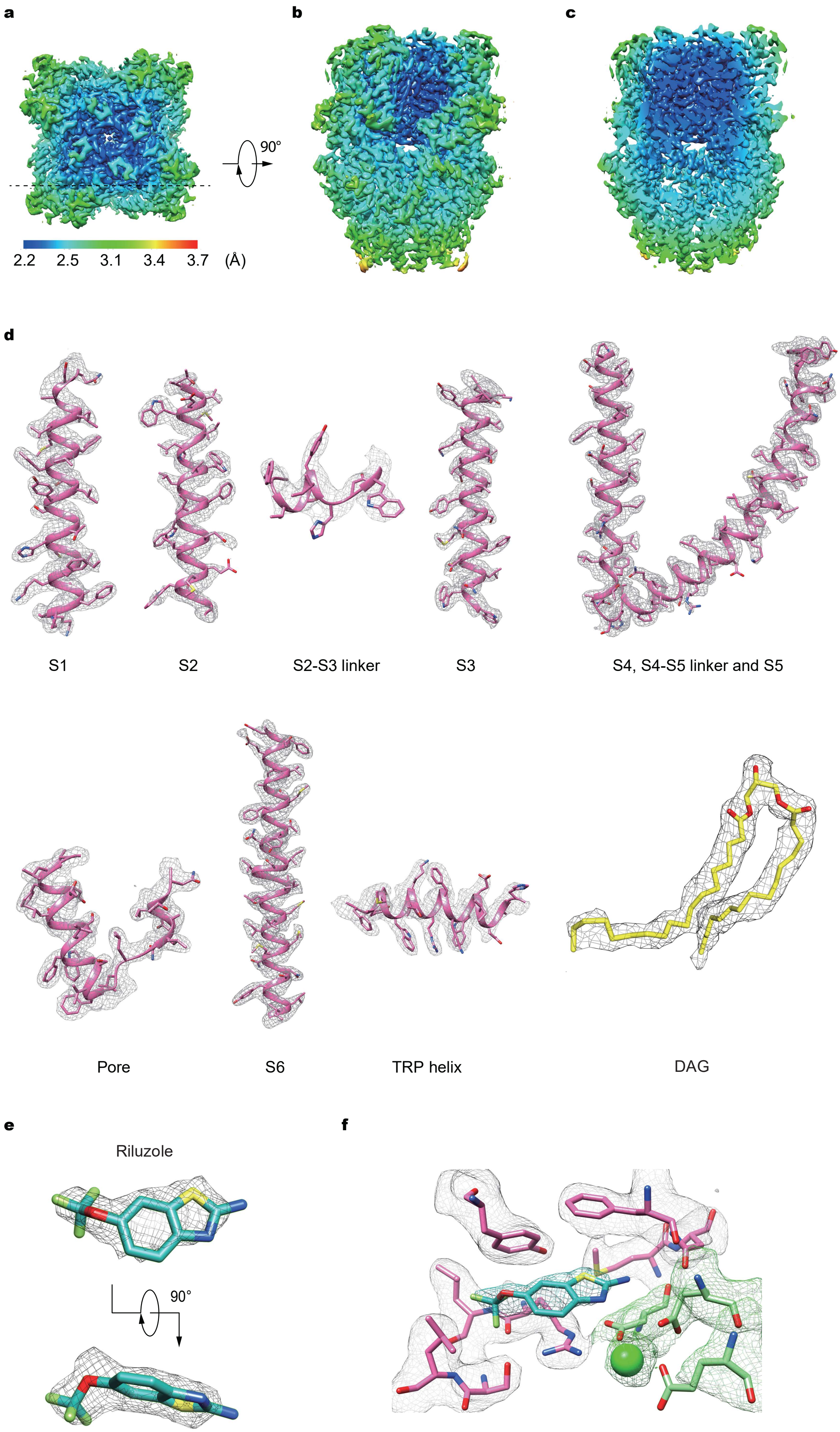

a
b
c
90°
2.2
2.5
3.1
3.4
3.7
(Å)
d
S1
S2
S2-S3 linker
S3
S4, S4-S5 linker and S5
Pore
S6
TRP helix
DAG
e
Riluzole
90°
f

Fig. S2

Supplementary Fig. S2. Cryo-EM map of riluzole-bound hTRPC5. a-c Cryo-EM map of riluzole-bound hTRPC5 colored by local resolution. Top view (**a**), side view (**b**) and cross-section view (**c**) are shown. The dashed line in (**a**) represents the position of the cross-section. **d** Cryo-EM density maps with superimposed atomic models for S1, S2, S2-S3 linker, S3, S4, S4-S5 linker, S5, pore, S6, TRP helix, and diacylglycerol (DAG). Density maps except DAG are shown in gray meshes (contoured at 2 σ). The density map of DAG is further sharpened by B factor of -28.4 $Å^2$ to show the density of oxygen on DAG. DAG is shown as sticks and colored in yellow. The maps are contoured at 3 σ and shown as gray mesh. **e** Density maps with superimposed atomic model for riluzole shown in side view (top) and bottom view (bottom). Riluzole is shown as sticks and colored in light sea green. The maps are contoured at 2.5 σ (gray mesh). **f** Densities of riluzole, Ca^{2+} ion, and nearby residues in hTRPC5. Riluzole-interacting residues and Ca^{2+}-interacting residues are colored in hot pink and light green, respectively. The maps of riluzole and Ca^{2+} ion are shown as light sea green meshes and green meshes, respectively. The maps of riluzole-interacting residues and Ca^{2+} ion-interacting residues are shown as gray meshes and light green meshes, respectively. The whole map is contoured at 2.5 σ.

Fig. S2

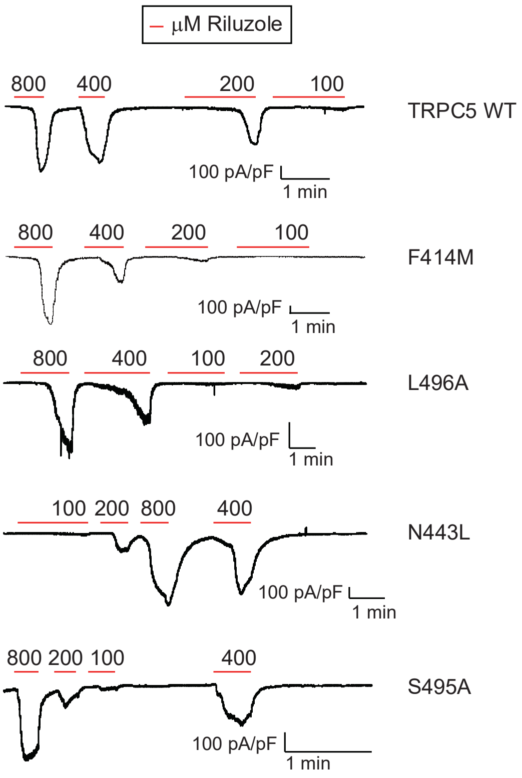

Supplementary Fig. S3. Representative traces for the potency of riluzole on various hTRPC5 constructs. HEK293 cells expressing hTRPC5 mutants were recorded in 0 mM extracellular Ca^{2+} with varying concentrations of riluzole. 100, 200, 400, and 800 represent the concentrations of riluzole at µM. Red lines represent the riluzole application.

Fig. S3

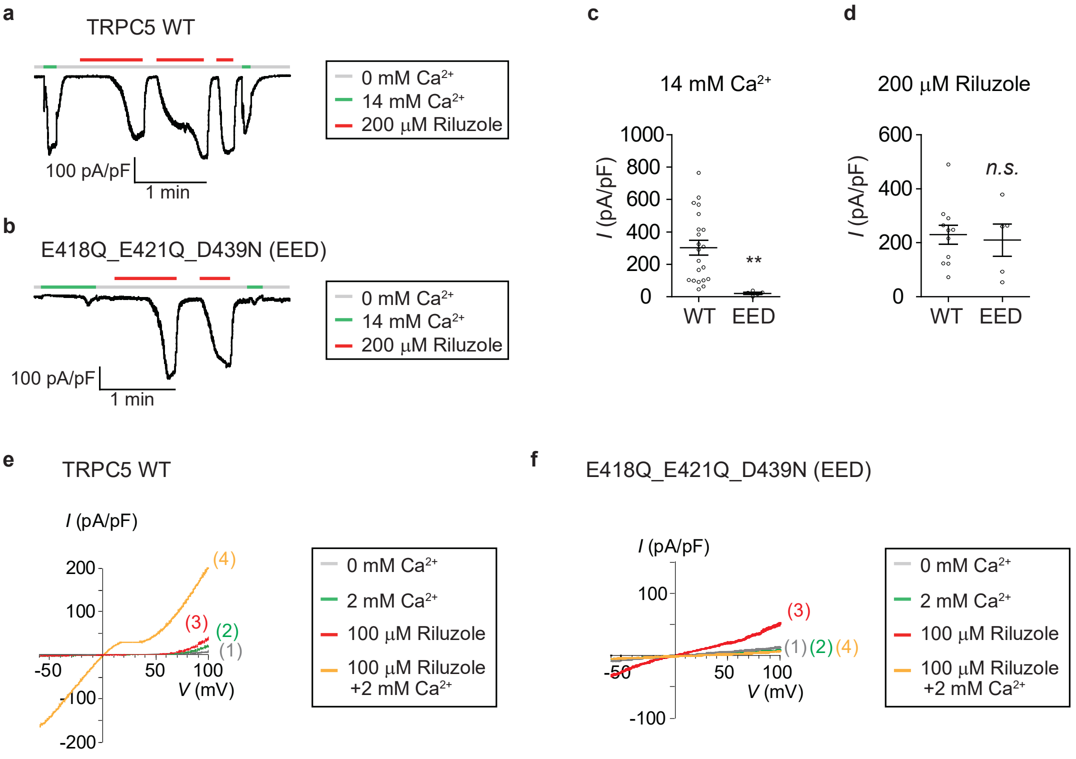

Supplementary Fig. S4. Ca^{2+}-dependent potentiation of riluzole-activated hTRPC5 currents. a, b Comparison of Ca^{2+} activation and riluzole activation between TRPC5 wild type (WT) (**a**) and TRPC5_E418Q_E421Q_D439N mutant (EED) (**b**). Representative currents of TRPC5 WT and EED mutant were elicited by 14 mM extracellular Ca^{2+} (marked as green lines to illustrate the periods of stimuli) and 200 µM riluzole in 0 mM Ca^{2+} (marked as red lines), respectively. Basal condition of 0 mM extracellular Ca^{2+} is marked as gray lines. **c, d** Statistical summaries of TRPC5 currents under 14 mM extracellular Ca^{2+} (**c**) or 200 µM riluzole (**d**) for TRPC5 WT and EED mutant. 5 or more than 5 cells were recorded for each condition. Data are shown in mean±SEM. Two-tailed unpaired Student's *t*-test was calculated with criteria of significance; **, $p<0.01$ and *n.s.* denotes for not significant ($p>0.05$). **e, f** Representative ramp traces of TRPC5 WT (**e**) and EED mutant (**f**) at basal state (0 mM Ca^{2+}, marked with (1) and colored in gray), 2 mM Ca^{2+} (2, in green), 100 µM riluzole in 0 mM Ca^{2+} (3, in red), and 100 µM riluzole in 2 mM Ca^{2+} (4, in yellow)..

Fig. S4

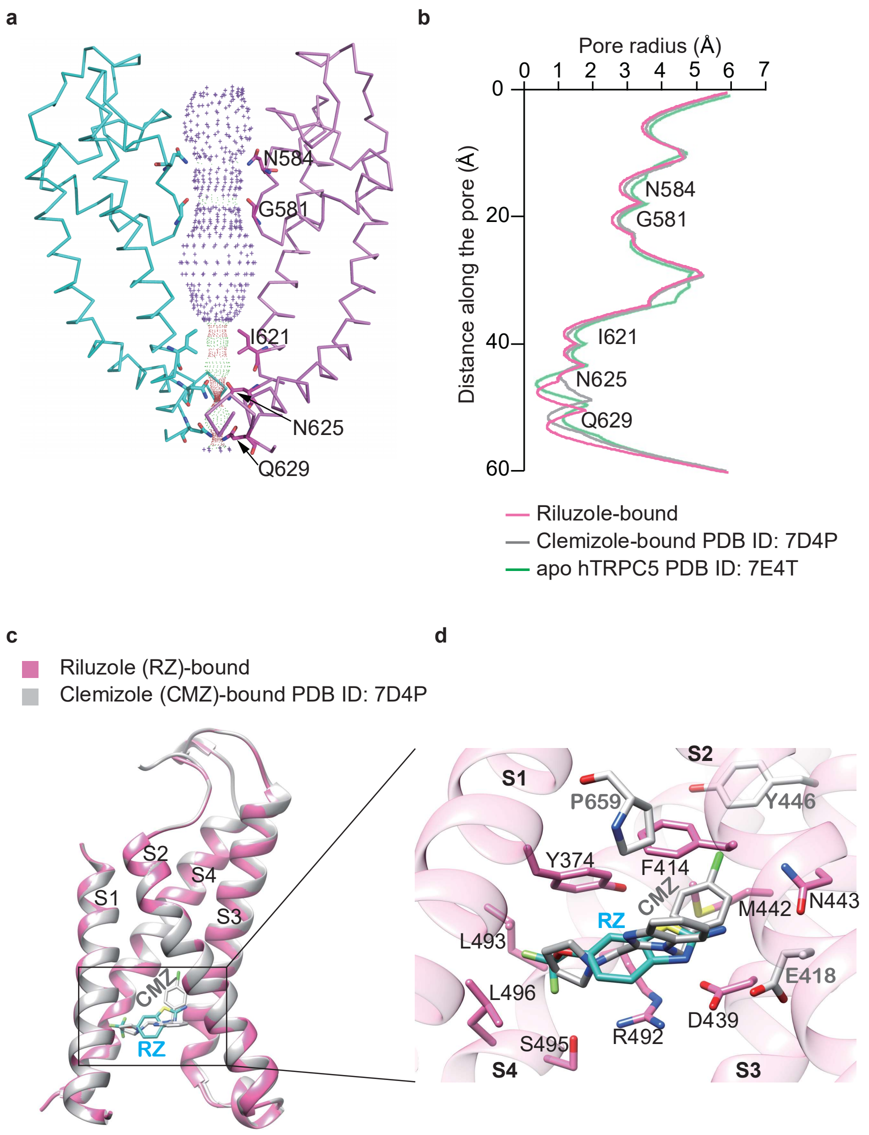

Supplementary Fig. S5. Structural comparison between riluzole-bound hTRPC5 and clemizole-bound hTRPC5. a Ion conduction pore in riluzole-bound hTRPC5. Two subunits of riluzole-hound hTRPC5 are shown as ribbon and colored in hot pink and cyan to highlight the ion conduction pathway (shown as dots) along the pore. Pore radii were calculated by HOLE. Purple, green and red dots represent pore radii of >2.8, 1.4-2.8, and <1.4 Å, respectively. **b** Profiles of calculated pore radii of riluzole-bound hTRPC5 are shown vertically and colored in hot pink. Profiles of apo hTRPC5 and clemizole-bound hTRPC5 are colored in green and gray, respectively. **c** Structural comparison between riluzole (RZ)-bound hTRPC5 and clemizole (CMZ)-bound hTRPC5 in S1-S4. The two structures are shown as cartoons, colored in hot pink and gray, respectively. Riluzole and clemizole situate at the identical pocket and are colored in light sea green and dark gray, respectively. **d** Close-up view of the drug-interacting residues in riluzole-bound hTRPC5 structure. The main chains of hTRPC5 are shown as cartoon and colored in transparent hot pink. Riluzole-interacting residues are shown as sticks and colored in hot pink. Residues of which interact with clemizole only are shown as sticks and colored in gray.

Fig. S5

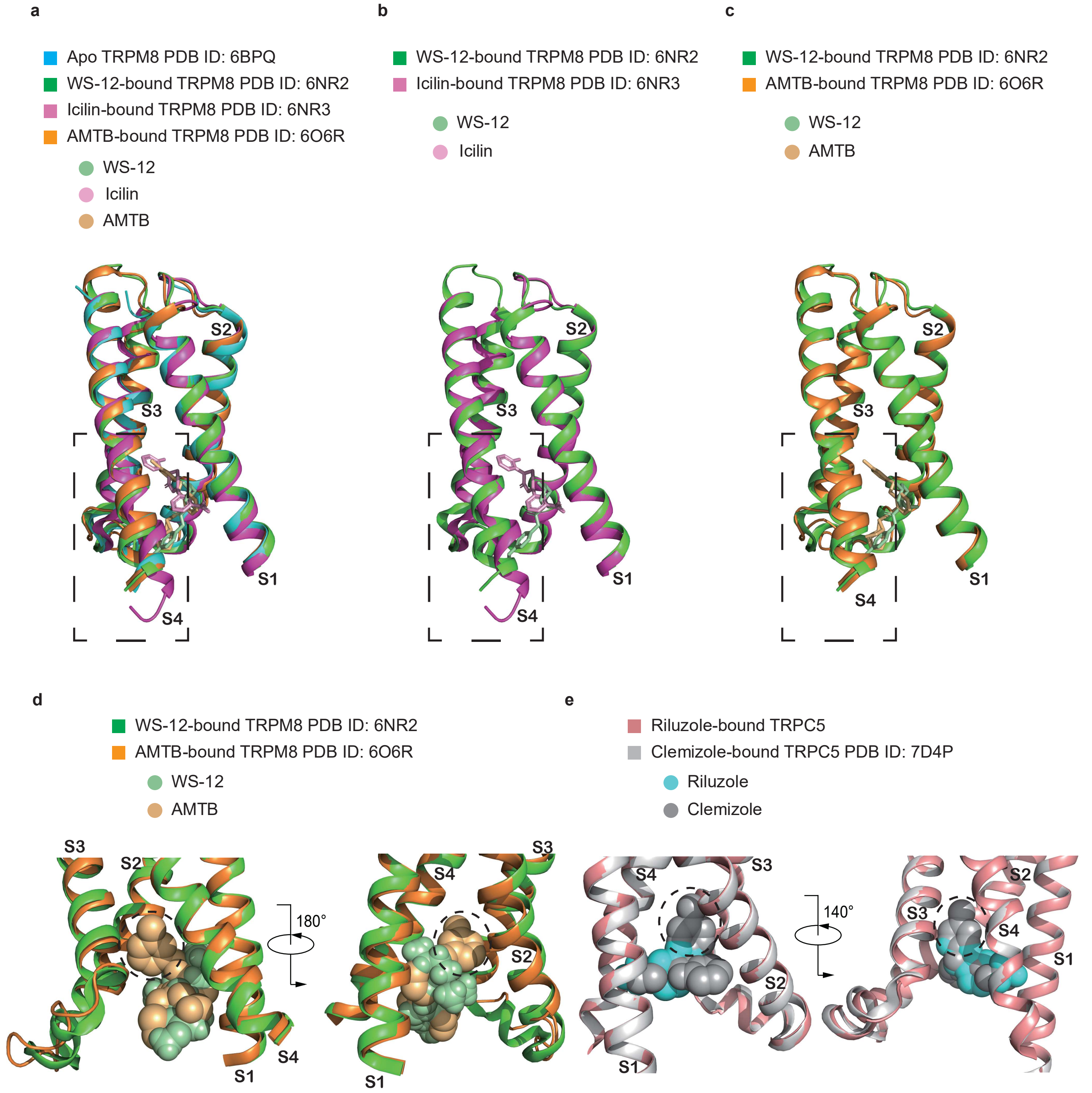

Supplementary Fig. S6. Structural comparisons of VSLD of TRPM8 and TRPC5. a-c Structural comparisons of VSLD among apo, WS-12 (TRPM8 agonist)-bound, icilin (TRPM8 agonist)-bound, and AMTB (TRPM8 antagonist)-bound TRPM8. Four VSLDs are shown as cartoons in side view, and colored as blue, green, magenta, and orange, respectively. WS-12, icilin, and AMTB are shown as sticks, and colored as light green, pink, and light orange, respectively. Comparisons of VLSD between WS-12-bound TRPM8 and icilin-bound TRPM8, or between WS-12-bound TRPM8 and AMTB-bound TRPM8 are shown in (**b**) or (**c**), respectively. Lower portions of S4 are boxed in dashes. The lower portion of S4 shows α helix in WS-12 or AMTB-bound TRPM8 but shows 3_{10} helix in icilin-bound TRPM8. **d, e** Structural comparisons of ligands in VLSD between WS-12-bound TRPM8 and AMTB-bound TRPM8 (**d**), or between riluzole-bound TRPC5 and clemizole-bound TRPC5 (**e**). Four structures are shown as cartoons, and colored as green, yellow, orange, and gray, respectively. Four ligands are shown as spheres, and colored as light green, light orange, cyan, and light gray, respectively. Two side views are shown. The antagonists, AMTB and clemizole, are larger than the agonists, WS-12 and riluzole, respectively. The extra van der Waals volumes of the two antagonists are circled by dashes.

Fig. S6

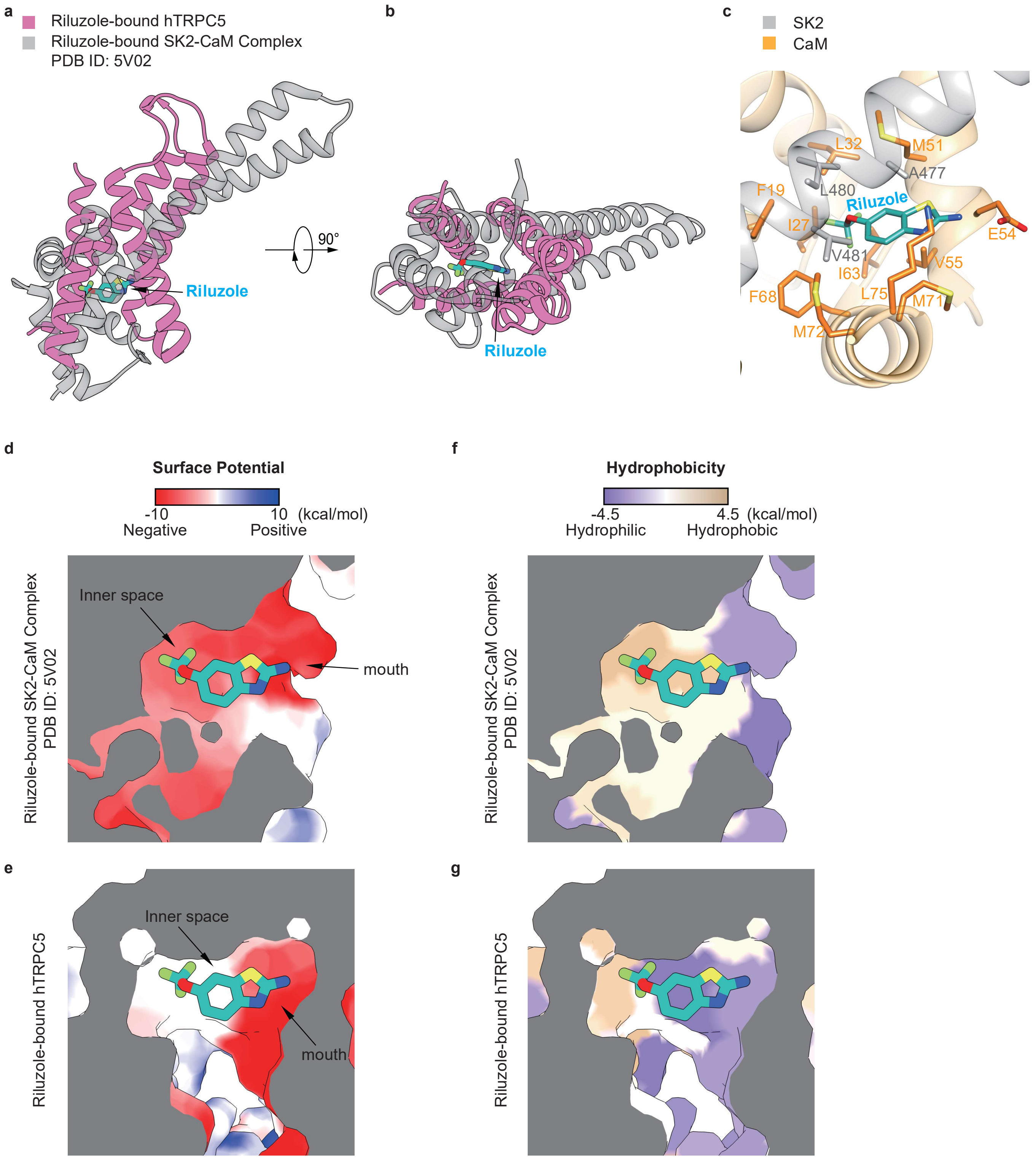

Supplementary Fig. S7. Surface analyses of riluzole-binding pockets in hTRPC5 and SK2-CaM complex. a, b Structural comparison of riluzole-binding pockets between hTRPC5 and SK2-CaM complex. Two structures are aligned by aligning riluzole. hTRPC5 and SK2-CaM complex are shown as cartoons and colored in hot-pink and semi-transparent gray, respectively. Riluzole is shown as sticks and colored in light sea green. **c** Close-up view of the riluzole-binding site in SK2-CaM complex. The main chains of SK2 and CaM are shown as cartoons and colored in semi-transparent gray and semi-transparent orange, respectively. The side chains of interacting residues from SK2 and CaM are shown as sticks, colored and labeled in gray and orange, respectively. **d, e** Surface analyses of electrostatic potential for riluzole-bound SK2-CaM complex (**d**) and riluzole-bound hTRPC5 (**e**) with gray-colored cross-sections, according to Coulomb's law. Negative, neutral, and positive potentials are colored in red, white, and blue, respectively. **f, g** Surface analyses of amino acid hydrophobicity for riluzole-bound SK2-CaM complex (**f**) and riluzole-bound hTRPC5 (**g**) with gray-colored cross-sections, according to the Kyte-Doolittle scale. The most polar, neutral, and most hydrophobic residues are colored in medium purple, white, and tan, respectively.

Fig. S7

Supplementary Table S1. Cryo-EM data collection, refinement, and validation statistics

	Riluzole-bound hTRPC5
PDB ID	7WDB
EMDB ID	EMD-32436
**Data collection and processing**	
Magnification	165,000 ×
Voltage (kV)	300
Electron exposure (e–/Å^2)	50
Defocus range (μm)	-1.5 to -2.0
Pixel size (Å)	0.821
Symmetry imposed	*C4*
Initial particle images (no.)	406,573
Final particle images (no.)	175,028
Map resolution (Å)	2.4
FSC threshold	0.143
Map resolution range (Å)	250-2.4
**Refinement**	
Initial model used (PDB code)	7d4p
Model resolution (Å)	250-2.4
FSC threshold	0.143
Model resolution range (Å)	250-2.4
Map sharpening *B* factor (Å^2)	-50
Model composition	
Non-hydrogen atoms	22,416
Protein residues	2,660
Ligands	28
B factors (Å^2)	
Protein	67.34
Ligand	71.44
R.m.s. deviations	
Bond lengths (Å)	0.005
Bond angles (°)	1.004
Validation	
MolProbity score	1.33
Clashscore	6.05
Poor rotamers (%)	0.84
Ramachandran plot	
Favored (%)	99.23
Allowed (%)	0.77
Disallowed (%)	0.00

Supplementary Table S1

Supplementary Table S2. The potency of riluzole on various human TRPC5 (hTRPC5) constructs.

hTRPC5 constructs	$LogEC_{50}$	Hill slope	EC_{50} (μM)
WT	2.34±0.05	3.69	221
F414M	2.77±0.04	2.70	583
N443L	2.45±0.02	3.41	281
S495A	2.58±0.15	2.79	384
L496A	2.42±0.03	5.23	262

Data are shown as mean±SEM. Recordings from at least 10 cells were analyzed for each mutant.
